# Associations between long-term night shift work and incidence of chronic obstructive pulmonary disease: a prospective cohort study of 277,059 UK Biobank participants

**DOI:** 10.1186/s12916-023-03240-8

**Published:** 2024-01-16

**Authors:** Jia Li, Liangle Yang, Yuxin Yao, Pei Gu, Yujia Xie, Haoyu Yin, Mingyue Xue, Yu Jiang, Jianghong Dai, Jixuan Ma

**Affiliations:** 1 Department of Radiology, Tongji Hospital, Tongji Medical College, Huazhong University of Science and Technology, Wuhan, 430030 Hubei China; 2 Key Laboratory of Environment and Health, Ministry of Education & Ministry of Environmental Protection, and State Key Laboratory of Environmental Health (Incubating), School of Public Health, Tongji Medical College, Huazhong University of Science and Technology, Wuhan, 430030 Hubei China; 3 Department of Occupational & Environmental Health, School of Public Health, Tongji Medical College, Huazhong University of Science and Technology, No.13 Hangkong Road, Wuhan, 430030 Hubei China; 4 Zane Cohen Centre for Digestive Diseases, Mount Sinai Hospital, Toronto, ON M5T3L9 Canada; 5 Department of Preventive Medicine, School of Public Health, Fujian Medical University, Fuzhou, 350122 China; 6 Department of Epidemiology and Biostatistics, School of Public Health, Xinjiang Medical University, Urumqi, 830017 China

**Keywords:** Night shift work, Genetic risk, COPD, Risk factor

## Abstract

**Background:**

Little is known about the effects of night shifts and their interactions with genetic factors on chronic obstructive pulmonary disease (COPD). In this study, we aim to investigate relationships between long-term night shift work exposure and COPD risk, and assess modification effects of genetic predisposition.

**Methods:**

A total of 277,059 subjects who were in paid employment or self-employed were included in the UK Biobank. Information on current and lifetime employment was obtained, and a weighted COPD-specific genetic risk score (GRS) was constructed. We used Cox proportional hazard models to investigate associations between night shift work and COPD risk, and their interaction with COPD-specific GRS.

**Results:**

The cohort study included 277,059 participants (133,063 men [48.03%]; mean [SD] age, 52.71 [7.08] years). During a median follow-up of 12.87 years, we documented 6558 incidents of COPD. From day work, irregular night shifts to regular night shifts, there was an increased trend in COPD incidence (*P* for trend < 0.001). Compared with day workers, the hazard ratio (HR) and 95% confidence interval (CI) of COPD was 1.28 (1.20, 1.37) for subjects with rarely/sometimes night shifts and 1.49 (1.35, 1.66) for those with permanent night shifts. Besides, the longer durations (especially in subjects with night shifts ≥ 10 years) and increasing monthly frequency of night shifts (in workers with > 8 nights/month) were associated with a higher COPD risk. Additionally, there was an additive interaction between night shifts and genetic susceptibility on the COPD risk. Subjects with permanent night shifts and high genetic risk had the highest risk of COPD (HR: 1.90 [95% CI: 1.63, 2.22]), with day workers with low genetic risk as a reference.

**Conclusions:**

Long-term night shift exposure is associated with a higher risk of COPD. Our findings suggest that decreasing the frequency and duration of night shifts may offer a promising approach to mitigating respiratory disease incidence in night shift workers, particularly in light of individual susceptibility.

**Supplementary Information:**

The online version contains supplementary material available at 10.1186/s12916-023-03240-8.

## Background

Chronic obstructive pulmonary disease (COPD) is a progressive disease characterized by consistent restriction of airflow along with varying degrees of obstructive bronchiolitis and parenchymal destruction [1–3]. It is estimated that over 350 million people have COPD worldwide, of whom about 3.2 million die each year, making it the third leading cause of mortality [4–6]. Although tobacco smoking has been considered the most important cause of COPD [7], recent evidence has indicated that approximately half of global COPD cases are thought to be caused by non-tobacco-related risk factors [1, 7]. Therefore, understanding pathogenesis, especially identifying novel risk factors contributing to COPD, should be emphasized.

With the progress of socioeconomic development in industrialized countries, shift work encompassing evening, night, and rotating shifts has become more prevalent worldwide [8, 9]. It is reported that approximately 15–20% of the working population in Europe and the USA are engaged in work that involves night shifts [10]. Since night shifts can result in a mismatch between actual and biological sleep times, disrupt circadian rhythm, impair sleep quality, and affect work-life balance [11–13], the harmful health effects of night shifts have become a growing concern in recent decades. A considerable number of studies indicated that the night shift was linked with an increased risk of cardiovascular diseases [13–15], metabolic disorders [11, 12, 16], infection susceptibility [17–20], and cancer [20–23]. Additionally, the circadian timing system drives daily changes in airway caliber, airway resistance, respiratory symptoms, mucus hypersecretion, and immune-inflammatory responses [24–26]. Therefore, we postulate that alteration in circadian patterns may result in airway damage and impaired lung function, ultimately contributing to the development of chronic airway diseases, such as COPD [24–30]. However, the relationships between long-term exposure to night shift work and COPD risk have not yet been addressed to date. Most importantly, no previous studies have documented the interaction effects of genetic susceptibility on relationships between night shifts on COPD incidence.

To fill these knowledge gaps, we used a dataset from the UK Biobank to investigate the relationships between long-term exposure to night shift work and COPD risk among 277,059 participants. In addition, we integrated information on in-depth lifetime employment to examine the impact of the duration and frequency of night shifts on COPD risk. For gene-environment interactions, we constructed a COPD-specific genetic risk score (GRS) to assess the interaction between genetic predisposition and night shifts on COPD risk.

## Methods

### Study design

The UK Biobank is a large-scale prospective cohort study that included > 500,000 community-dwelling adults from the UK between 2006 and 2010 [31]. The study design and the protocol of the UK Biobank have been described in detail elsewhere [31].

**Fig. 1:**
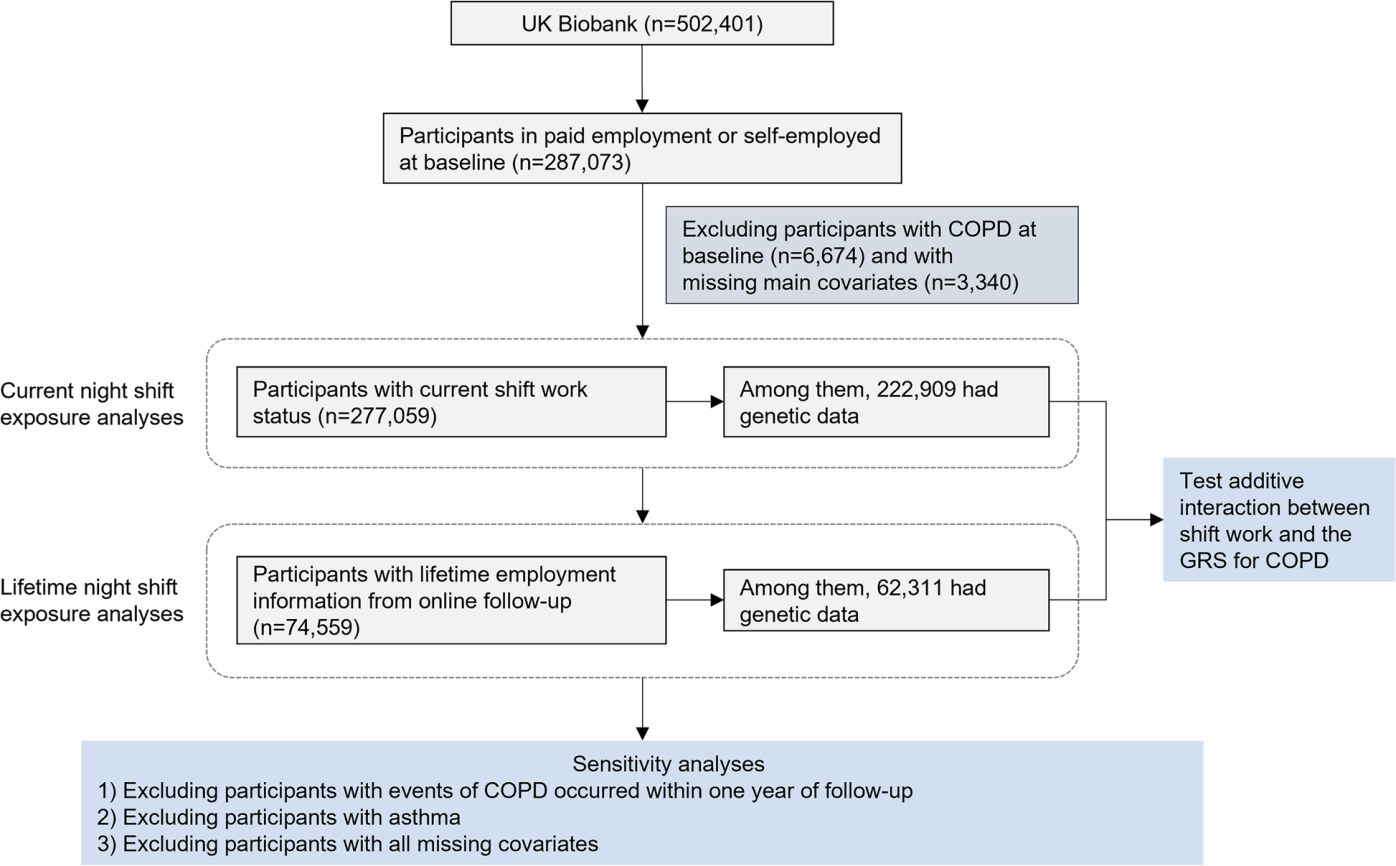
Flowchart of the study. *GRS* Genetic risk score, *COPD* Chronic obstructive pulmonary disease

In this study, our analyses focused on participants who were in paid employment or self-employed (*n* = 287,073). Among them, subjects with missing covariates (including age, body mass index [BMI], Townsend deprivation index, smoking status, alcohol drinking status, and sleep duration [*n* = 3340]) were excluded. For the analysis of incident COPD, we also excluded subjects with COPD at baseline (*n* = 6674). Overall, 277,059 participants were analyzed in the primary analysis, and 222,909 European subjects were included in the subsequent gene-environment interaction analysis. Out of those, 74,559 subjects have complete lifetime employment information from the follow-up in 2015 through online questionnaires [32], and a subgroup of 62,311 participants are available for genetic analysis (Fig. 1).

### Shift work assessment

Each participant received an employment information survey, in which they were asked whether their current employment involved shift work. If the answer was yes, subjects were further inquired to choose whether the job involved night shifts, which means a work schedule that involves work through the normal sleeping hours (from 00:00 to 06:00). Based on this information and previous studies [14, 16], we categorized all subjects into “day workers,” “shift, but rarely/some night shifts,” and “usual/permanent night shifts”.

In the assessment of lifetime employment, subjects reported each job ever worked, the number of years in each job, and the number of night shifts per month each job entailed [16]. The detailed assessment method has been described in previous studies [14–16]. Based on the above information, we calculated the lifetime duration (years) and average frequency (number/per month) of night shifts for each participant [14–16].

### Outcome ascertainment

Prevalent cases of COPD were derived by using self-reported information, primary care data, death register records, and hospital admission data through linkage to the UK National Health Services register with the International Classification of Diseases Version 10 (ICD-10) codes as J40-J44 [33]. To avoid the interference of asthma [34], we further collected information on asthma diagnosis, which was coded as J45 based on the ICD-10. In this study, COPD incidence refers to newly diagnosed cases.

### Genetic risk score (GRS) construction

The explicit procedure of the genotype, quality control, and imputation of the UK Biobank study has been well described before [35]. Based on a recent genome-wide association study [36], we selected 22 independent single nucleotide polymorphisms (SNPs) to construct the weighted COPD-specific GRS for each individual. The details about the selected SNPs are provided in Additional file 1: Table S1. The weighted GRS was calculated by using the summing formula [36]:

$$\mathrm{GRS }= ({\beta }_{1}\hspace{0.17em}\times \hspace{0.17em}{{\text{SNP}}}_{1}\hspace{0.17em}+\hspace{0.17em}{\beta }_{2}\hspace{0.17em}\times \hspace{0.17em}{{\text{SNP}}}_{2}\hspace{0.17em}+\hspace{0.17em}\dots \hspace{0.17em}+{\hspace{0.17em}\beta }_{22}\hspace{0.17em}\times \hspace{0.17em}{{\text{SNP}}}_{22})\hspace{0.17em}\times \hspace{0.17em}(22/\mathrm{sum\;of\;the\;}\beta \mathrm{\;coefficients})$$

### Covariates

Demographic data including age, sex, ethnicity (white/others), BMI, the Townsend deprivation index, sleep duration (hours), smoking status, alcohol drinking status, physical activity, and chronotype were collected. Chronotype was categorized as morning type, intermediate type, and evening type [14]. As the proportion of participants choosing “do not know,” “prefer not to answer,” or did not answer chronotype was over 10%, we classified these populations as “not reported” in the primary analysis and then excluded them in the sensitivity analyses. Physical activity was assessed by the validated Short International Physical Activity Questionnaire (IPAQ), and the final scores were divided into three levels of physical activity (“low,” “moderate,” and “high”) [15]. Similarly, the rest of the participants were grouped as “not reported” in the primary analysis and were excluded in the following sensitivity analyses.

### Statistical analyses

We used Cox proportional hazard models to estimate the association between night shift work and the risk of incident COPD, with the results reported as hazard ratios (HRs) and 95% confidence intervals (CIs). Person years were aggregated from baseline until the date of death or diagnosis, loss of follow-up, or end of follow-up, whichever occurred first. In the primary analysis, model 1 showed crude associations, while age and sex were adjusted in model 2. Model 3 additionally included ethnicity, BMI, Townsend deprivation index, sleep duration, smoking status, alcohol drinking, IPAQ activity group, and chronotype. Model 4 included asthma in addition to covariates in model 3. In all models, “day workers” were set as the reference. We also performed stratified analyses by sex, age, BMI, and chronotype to investigate associations of night shift work with COPD risk in different socioeconomic subgroups.

In addition, we used Cox proportional hazards models to estimate the constructed GRS in relation to COPD incidence, and the restricted cubic spline model (RCS) was further applied to examine the dose–response relationships. To test whether genetic risk modified the effect of night shifts on the risk of incident COPD, we first divided participants into subgroups by genetic risk (low vs. high) based on the median value of COPD-specified GRS. Then, we included a product term of COPD-specific GRS (low vs. high) with their current shift work status or lifetime night shift work duration or average monthly frequency of night shifts in the models, respectively. In the above calculations, “day workers” with low genetic risk were set as the reference. We further used the relative excess risk to interaction (RERI) and the attributable proportion (AP) as the measure of interaction on the additive scale.

To examine the robustness of the results, we carried out the following sensitivity analyses: (1) excluding incident cases that occurred within the first year, (2) excluding participants with asthma, (3) excluding participants with all missing covariates.

All analyses were performed using R software, version 4.2.1 (R Foundation for Statistical Computing). All *P* values were two-sided, and *P* < 0.05 was considered to indicate statistical significance.

## Results

Among 277,059 subjects, the proportions of “day workers,” “shift, but rarely/some night shifts,” and “usual/permanent night shifts” were 82.83%, 13.37%, and 3.80%, respectively (Table 1). Night shift workers were more likely to be male, younger, current smokers, and have higher BMI and shorter sleep duration in comparison to day workers. The baseline characteristics of subjects according to the monthly frequency and duration of night shifts are presented in Additional file 1: Table S2-3. The comparison of baseline characteristics in the participants with or without genetic data is shown in Additional file 1: Table S4.

**Table 1:** Basic characteristics of participants by current work schedule (*n* = 277,059)

Characteristics	Current work schedule			*P* value
	**Day workers**	**Shift, but rarely/some night shifts**	**Usual/permanent night shifts**	
No. of subjects (%)	229,478 (82.83)	37,057 (13.37)	10,524 (3.80)	
Male, No. (%)	106,946 (46.60)	19,563 (52.79)	6554 (62.28)	< 0.001
Age, mean (SD), years	52.89 ± 7.09	51.96 ± 7.00	51.23 ± 6.79	< 0.001
BMI, mean (SD), kg/m^2^	27.07 ± 4.63	27.92 ± 4.94	28.41 ± 4.89	< 0.001
White, no. (%)	217,873 (94.94)	33,153 (89.46)	9126 (86.72)	< 0.001
Smoking status, No. (%)				< 0.001
Never	134,324 (58.53)	19,976 (53.91)	5608 (53.29)	
Previous	73,077 (31.84)	11,696 (31.56)	3152 (29.95)	
Current	22,077 (9.62)	5385 (14.53)	1764 (16.76)	
Alcohol drinking, No. (%)				< 0.001
Never	6992 (3.05)	1779 (4.80)	643 (6.11)	
Previous	5783 (2.52)	1279 (3.45)	399 (3.79)	
Current	216,703 (94.43)	33,999 (91.75)	9482 (90.10)	
Sleep duration, mean (SD), h/day	7.07 ± 0.94	6.97 ± 1.06	6.83 ± 1.18	< 0.001
Townsend deprivation index, median (IQR)	− 2.26 (− 3.70 to 0.17)	− 1.34 (− 3.20 to 1.62)	− 1.12 (− 3.09 to 1.95)	< 0.001
IPAQ activity group, No. (%)				< 0.001
Low	39,990 (17.43)	4385 (11.83)	1057 (10.04)	
Moderate	79,622 (34.70)	9849 (26.58)	2434 (23.13)	
High	71,981 (31.37)	14,866 (40.12)	4503 (42.79)	
Not reported	37,885 (16.51)	7957 (21.47)	2530 (24.04)	
Chronotype, No. (%)				< 0.001
Morning type	53,044 (23.12)	9158 (24.71)	1987 (18.88)	
Intermediate type	133,571 (58.21)	20,585 (55.55)	5445 (51.74)	
Evening type	18,203 (7.93)	3096 (8.35)	1566 (14.88)	
Not reported	24,660 (10.75)	4218 (11.38)	1526 (14.50)	
Asthma, No. (%)	29,970 (13.06)	5004 (13.50)	1370 (13.02)	0.06

*Abbreviations*: *BMI* Body mass index, *IPAQ* International Physical Activity Questionnaire

Values are given as mean ± standard deviation or median (interquartile range) for continuous variables and percentage for categorical variables. Percentages have been rounded and therefore may not total 100

*P* for values of continuous variables if distributed normally were estimated by one-way ANOVA test, otherwise Kruskal–Wallis test

*P* for values of categorical variables were estimated by chi-square test

During a median of 12.87 years of follow-up, we documented 6558 incidents of COPD. In fully adjusted models (Table 2, model 4), we observed that night shift work was significantly related to a higher risk of COPD (all *P* for trend < 0.001). Compared with day workers, the HR (95% CI) of COPD was 1.28 (1.20, 1.37) in shift workers who rarely or sometimes undertook night shifts and 1.49 (1.35, 1.66) in those with regular night shift work. When we excluded COPD cases within the first year (Additional file 1: Table S5), asthma (Additional file 1: Table S6), or all missing covariates (Additional file 1: Table S7), the effect estimates were consistent with the main analyses. Stratified analyses further demonstrated that the observed associations seem to be more pronounced among females, individuals with age ≥ 60 years, and those with BMI < 25 kg/m^2^ (Additional file 1: Table S8), although the interaction effects were statistically non-significant (*P* for interaction > 0.05). We further analyzed the relationships between chronotype and COPD risk in this study population. We observed that subjects who were evening persons had a higher risk of COPD compared with those describing themselves as intermediate chronotypes. The HR (95% CI) for COPD in those reporting being an evening person was 1.09 (1.01, 1.19) in fully adjusted models (Additional file 1: Table S9).

**Table 2:** Associations between current night shift work and the risk of incident COPD (*n* = 277,059)

Variables	HR (95% CI) by current work schedule			*P* for trend
	**Day workers**	**Shift, but rarely/some night shifts**	**Usual/permanent night shifts**	
Case/all subjects	4984/229,478	1177/37,057	397/10,524	
Model 1	1 [reference]	1.48 (1.38, 1.57)	1.75 (1.58, 1.94)	< 0.001
Model 2	1 [reference]	1.60 (1.50, 1.70)	2.02 (1.82, 2.24)	< 0.001
Model 3	1 [reference]	1.29 (1.21, 1.37)	1.47 (1.32, 1.63)	< 0.001
Model 4	1 [reference]	1.28 (1.20, 1.37)	1.49 (1.35, 1.66)	< 0.001

*Abbreviations*: *COPD* Chronic obstructive pulmonary disease, *BMI* Body mass index, *IPAQ* International Physical Activity Questionnaire

Model 1: Unadjusted

Model 2: Model 1 + age and sex

Model 3: Model 2 + ethnicity, BMI, Townsend deprivation index, sleep duration, smoking status, alcohol drinking, IPAQ activity group, and chronotype

Model 4: Model 3 + asthma

In fully adjusted models, we observed that the longer durations of night shift work were associated with a higher risk of COPD (Table 3, model 4). Compared with day workers, the HR (95% CI) of COPD was 1.17 (1.00, 1.38) among subjects reporting < 10 years of night shifts; and the higher risk was found in subjects who underwent night shift work over 10 years (HR 1.23 [95% CI 1.03, 1.46]). In addition, we observed that workers who undertook > 8 nights/month had the highest COPD risk (HR 1.41 [95% CI 1.19, 1.68]) (Table 4). In sensitivity analyses, all the above results were consistent with the main analyses (Additional file 1: Table S5-7).

**Table 3:** Associations between lifetime duration of night shift work and the risk of incident COPD (*n* = 74,559)

Variables	HR (95% CI) by lifetime duration of night shift work			*P* for trend
	**None**	** < 10 years**	** ≥ 10 years**	
Case/all subjects	805/56,698	176/9673	164/8188	
Model 1	1 [reference]	1.28 (1.09, 1.51)	1.42 (1.20, 1.67)	< 0.001
Model 2	1 [reference]	1.33 (1.13, 1.57)	1.48 (1.25, 1.75)	< 0.001
Model 3	1 [reference]	1.18 (1.00, 1.39)	1.22 (1.03, 1.45)	0.007
Model 4	1 [reference]	1.17 (1.00, 1.38)	1.23 (1.03, 1.46)	0.007

*Abbreviations*: *COPD* Chronic obstructive pulmonary disease, *BMI* Body mass index, *IPAQ* International Physical Activity Questionnaire

Model 1: Unadjusted

Model 2: Model 1 + age and sex

Model 3: Model 2 + ethnicity, BMI, Townsend deprivation index, sleep duration, smoking status, alcohol drinking, IPAQ activity group, and chronotype

Model 4: Model 3 + asthma

**Table 4:** Associations between average lifetime frequency of night shifts worked and the risk of incident COPD (*n* = 74,559)

Variables	HR (95% CI) by average lifetime night shift frequency				***P* for trend**
	**None**	** < 3/month**	**3–8/month**	** > 8/month**	
Case/all subjects	805/56,698	49/2381	125/8919	166/6561	
Model 1	1 [reference]	1.46 (1.09, 1.94)	0.99 (0.82, 1.19)	1.79 (1.51, 2.11)	< 0.001
Model 2	1 [reference]	1.43 (1.07, 1.91)	1.08 (0.90, 1.31)	1.78 (1.50, 2.10)	< 0.001
Model 3	1 [reference]	1.30 (0.97, 1.73)	0.98 (0.81, 1.18)	1.42 (1.20, 1.69)	0.001
Model 4	1 [reference]	1.29 (0.97, 1.73)	0.98 (0.81, 1.18)	1.41 (1.19, 1.68)	0.001

*Abbreviations*: *COPD* Chronic obstructive pulmonary disease, *BMI* Body mass index, *IPAQ* International Physical Activity Questionnaire

Model 1: Unadjusted

Model 2: Model 1 + age and sex

Model 3: Model 2 + ethnicity, BMI, Townsend deprivation index, sleep duration, smoking status, alcohol drinking, IPAQ activity group, and chronotype

Model 4: Model 3 + asthma

**Fig. 2:**
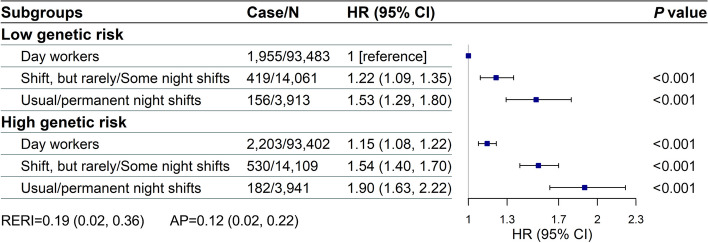
Joint effects of genetic risk score (GRS) with current work schedule on COPD risk. *AP* Attributable proportion, *COPD* Chronic obstructive pulmonary disease, *HR* hazard ratio, *CI* Confidence interval, *BMI* Body mass index, *IPAQ* International Physical Activity Questionnaire. *RERI* Relative excess risk to interaction. Adjusted for age, sex, BMI, Townsend deprivation index, sleep duration, smoking status, alcohol drinking, IPAQ activity group, chronotype, and asthma

We first noted that higher GRS was significantly and positively related to higher COPD risk in dose–response manners. Each SD increment in GRS was associated with a 9% increase in COPD risk (HR 1.09 [95% CI 1.06, 1.12]) (Additional file 1: Table S10; Fig. S1). Furthermore, we observed significant joint effects between genetic risk and current work schedule on the COPD risk; the overall risk of COPD incidence increased as both genetic risk and night work-related risk increased (Fig. 2). Compared with day workers who had lower genetic risk, individuals with usual/permanent night shift work and high genetic risk had the highest risk of COPD (HR 1.90 [95% CI 1.63, 2.22]). The RERI and AP for interaction between the current work schedule and genetic risk were 0.19 (0.02, 0.36) and 0.12 (0.02, 0.22), respectively. In addition, we also found the cumulative effects between the duration of night shift work and genetic risk on COPD risk (Additional file 1: Fig. S2). Compared with day workers with low genetic risk, subjects with night shift work over 10 years and high genetic risk showed the highest risk of COPD (HR 1.30 [95% CI 1.00, 1.67]) (Additional file 1: Fig. S2). Similarly, we demonstrated significant joint effects of the monthly frequency of night shift work and genetic risk on COPD risk. Workers who undertook over 8 nights/month and high genetic risk had higher COPD risk (HR 1.55 [95% CI 1.21, 1.99]), with day workers with low genetic risk as the reference (Additional file 1: Fig. S3).

## Discussion

In this study, we observed that night shifts were significantly associated with an increased risk of COPD. From day work, irregular night shifts to regular night shifts, there was an elevated trend in COPD incidence. Besides, longer durations of night shift work were related to a higher COPD risk. Regarding the frequency of night shifts, we found that the increasing monthly frequency of night shifts was positively related to COPD risk. In particular, there was a significant additive interaction between genetic susceptibility and night shifts. Participants with usual/permanent night shift work and high genetic risk had the highest risk of incident COPD compared to those with day workers who had lower genetic risk.

To the best of our knowledge, this is the first prospective cohort study to investigate the relationships between night shift work and the risk of incident COPD. Although existing literature has suggested that night shift work is related to a number of serious health conditions, such as cardiovascular diseases [13–15], metabolic disorders [11, 12, 16], increased infection susceptibility [17–20], and cancer [20–23], the adverse effects of the night shifts on the respiratory system have been rarely mentioned. Only a limited number of studies reported that shift work was related to declined lung function [34, 37], higher odds of asthma [34], and increased risk of lung cancer [38, 39]. To some extent, our results may be partly supported by those findings. For example, Maidstone et al. proposed that individuals with night shifts may suffer a greater risk of developing asthma in comparison to those day workers [34]. They further observed that workers with permanent night shifts experienced a significant decline in lung function [34]. Consistently, Nemery et al. found that lung function was unchanged over the morning shift among steelworkers, but those spirometry indices had a pronounced decrease in those performing night shift work [37]. Additionally, Schernhammer et al. reported an increased risk of lung cancer among women who worked rotating night shifts [38]. In this study, we observed that the night shift was a noteworthy and new risk factor for COPD. These findings not only expand our understanding of the harmful respiratory effects of night shifts but also provide valuable evidence for the development of primary prevention strategies for COPD. Besides, we observed that longer durations and monthly frequency of night shift work were important determinants of increased risk of COPD. Similar to our findings, Schernhammer et al. enrolled 78,612 women in the Nurses’ Health Study and demonstrated that the increasing years of night shifts was positively related to lung cancer risk [38]. Maidstone et al. suggested that more frequent nights could contribute to a higher risk of asthma [34]. All these findings provide evidence on detrimental relationships between night shift work and health and give a clue that reducing the frequency and duration of night shift work might be an effective measure to prevent respiratory diseases in night shift workers.

There are several potential mechanisms underlying the associations between night shift work and COPD risk. Night shifts may trigger circadian disruption, resulting from the mismatch between internal circadian rhythms and external environmental conditions [40]. Subsequently, the changes in sleep–wake cycles and exposure to light–dark patterns can lead to abnormal levels of cortisol, melatonin, and body temperature, which have been associated with COPD [40–43]. For example, the night shift can alter the circadian rhythm, which has been reported to suppress melatonin biosynthesis [15, 44, 45]. Previous experimental studies have shown that melatonin has a beneficial effect on COPD by attenuating apoptosis and endoplasmic reticulum stress [42]. Therefore, lower melatonin levels caused by disruption of circadian rhythm may be an important pathway that contributes to the observed associations. Additionally, circadian disruption induced by night shifts can disrupt the normal expression of clock gene expression, such as *BMAL*, *CLOCK, PER1*, *PER2*, and *PER3* [46]. The oscillation and dysregulated expression of these clock gene expression have been reported to play a role in COPD pathogenesis, including chronic inflammation and imbalanced autophagy level [47]. On the other hand, circadian clock disruption during chronic lung diseases has essential effect in augmented oxidative stress and increased systemic inflammation, as evidenced by increased interleukin-6, C-reactive protein, and tumor necrosis factor [14, 48]. These factors are believed to be responsible for the advancement of COPD [49]. Lastly, unfavorable changes in health behaviors (such as increased smoking and irregular meals), among night shift workers, could also be a potential reason that contributes to COPD.

Significant additive interactions between genetic predisposition and current shift work were observed in the current study. These results support public health efforts to emphasize the harmful impact of night shift work, especially for those individuals who are at high genetic risk. We also observed that the risk of COPD with longer durations and increased monthly frequency was aggravated by high genetic risk, despite the lack of statistical significance in the interaction test. All findings might facilitate the emergence of new strategies for personalized precision prevention of COPD and highlight that further primary prevention strategies of COPD for night shift workers should take into account individual susceptibility.

Strength of our study lies in the prospective design, large sample size, long-term follow-up, and detailed current shift work information. Most importantly, this is the first cohort study to investigate relationships between night shifts and COPD risk. Besides, we further assessed the contribution of genetic factors in the above relationships, which enabled us to precisely determine the effects of night shifts on groups with varying levels of susceptibility. Moreover, we used detailed information on the lifetime of night shift work, which provides original insight and a unique opportunity to study the relationship between night shifts and COPD [14].

Nevertheless, several potential weaknesses also exist in this study. First, causality cannot be determined because of the observational study design. Second, the information on current and former employment history and some of the COPD incidence was assessed by self-report, which may contribute to misclassification. Third, we did not assess the role of melatonin in the observed relationships, thus we could not confirm our findings mechanistically. Fourth, most of the participants in this study were white, which limits the generalizability of our results to other racial/ethnic groups. Lastly, online lifetime employment information only enrolled ~ 70,000 individuals in this study.

## Conclusions

In conclusion, night shift work was significantly associated with an increased risk of COPD and maybe a novel risk factor for COPD. Besides, we found that the positive relationships between night shift work and COPD risk could be aggravated by higher COPD-genetic risk.

### Supplementary Information

**Additional file 1: Table S1.** Information on genetic variants associated with COPD. **Table S2.** Basic characteristics of participants by lifetime duration of night shift work exposure. **Table S3.** Basic characteristics of participants by average lifetime number of night shift work exposure. **Table S4.** Basic characteristics between all subjects and those with genetic data. **Table S5.** Adjusted HRs (95% CIs) of the risk of incident COPD by current work schedule and lifetime night shift work experience after excluding participants with COPD occurred within 1 year of follow-up. **Table S6.** Adjusted HRs (95% CIs) of the risk of incident COPD by current work schedule and lifetime night shift work experience after excluding participants with asthma. **Table S7. **Adjusted HRs (95% CIs) of the risk of incident COPD by current work schedule and lifetime night shift work experience after excluding participants with all missing covariates. **Table S8.** Associations of current shift work schedule and the risk of incident COPD stratified by sex, age, BMI, and chronotype. **Table S9.** Associations between chronotype and the risk of incident COPD. **Table S10.** Adjusted HRs (95% CIs) of incident COPD by individual genetic risk score (GRS) of participants of current work schedule. **Fig. S1.** Association of genetic risk score (GRS) and the risk of incident COPD (*n*=222,909). **Fig. S2.** Joint effects of genetic risk score (GRS) with lifetime duration of night shift work exposure on the risk of incident COPD (*n*=62,311). **Fig. S3.** Joint effects of genetic risk score (GRS) with average lifetime number of night shift work exposure on the risk of incident COPD (*n*=62,311).

## Data Availability

The UK Biobank resources are available from the authors upon reasonable request and can be accessed through applications on their website (https://www.ukbiobank.ac.uk/).

## Abbreviations

AP: Attributable proportion
BMI: Body mass index
COPD: Chronic obstructive pulmonary disease
CI: Confidence interval
GRS: Genetic risk score
HR: Hazard ratios
IPAQ: International Physical Activity Questionnaire
ICD-10: International Classification of Diseases Version 10
RCS: Restricted cubic spline
RERI: Relative excess risk to interaction
SNPs: Single nucleotide polymorphisms
