# Supplementary material for: Associations between long-term night shift work and incidence of chronic obstructive pulmonary disease: a prospective cohort study of 277,059 UK Biobank participants
Source: BMC Med. 2024 Jan 16;22:16. doi: 10.1186/s12916-023-03240-8 (PMC10790498; doi:10.1186/s12916-023-03240-8)
Supplement: Supplementary file 1 — Additional file 1: Table S1. Information on genetic variants associated with COPD. Table S2. Basic characteristics of participants by lifetime duration of night shift work exposure. Table S3. Basic characteristics of participants by average lifetime number of night shift work exposure. Table S4. Basic characteristics between all subjects and those with genetic data. Table S5. Adjusted HRs (95% CIs) of the risk of incident COPD by current work schedule and lifetime night shift work experience after excluding participants with COPD occurred within 1 year of follow-up. Table S6. Adjusted HRs (95% CIs) of the risk of incident COPD by current work schedule and lifetime night shift work experience after excluding participants with asthma. Table S7. Adjusted HRs (95% CIs) of the risk of incident COPD by current work schedule and lifetime night shift work experience after excluding participants with all missing covariates. Table S8. Associations of current shift work schedule and the risk of incident COPD stratified by sex, age, BMI, and chronotype. Table S9. Associations between chronotype and the risk of incident COPD. Table S10. Adjusted HRs (95% CIs) of incident COPD by individual genetic risk score (GRS) of participants of current work schedule. Fig. S1. Association of genetic risk score (GRS) and the risk of incident COPD (n=222,909). Fig. S2. Joint effects of genetic risk score (GRS) with lifetime duration of night shift work exposure on the risk of incident COPD (n=62,311). Fig. S3. Joint effects of genetic risk score (GRS) with average lifetime number of night shift work exposure on the risk of incident COPD (n=62,311). [file 12916_2023_3240_MOESM1_ESM.docx]

**Additional file 1**

**Table S1.** Information on genetic variants associated with COPD.

**Table S2.** Basic characteristics of participants by lifetime duration of night shift work exposure.

**Table S3.** Basic characteristics of participants by average lifetime number of night shift work exposure.

**Table S4.** Basic characteristics between all subjects and those with genetic data.

**Table S5.** Adjusted HRs (95% CIs) of the risk of incident COPD by current work schedule and lifetime night shift work experience after excluding participants with COPD occurred within 1 year of follow-up.

**Table S6.** Adjusted HRs (95% CIs) of the risk of incident COPD by current work schedule and lifetime night shift work experience after excluding participants with asthma.

**Table S7.** Adjusted HRs (95% CIs) of the risk of incident COPD by current work schedule and lifetime night shift work experience after excluding participants with all missing covariates.

**Table S8.** Associations of current shift work schedule and the risk of incident COPD stratified by sex, age, BMI, and chronotype.

**Table S9.** Associations between chronotype and the risk of incident COPD.

**Table S10.** Adjusted HRs (95% CIs) of incident COPD by individual genetic risk score (GRS) of participants of current work schedule.

**Fig. S1.** Association of genetic risk score (GRS) and the risk of incident COPD (n=222,909).

**Fig. S2.** Joint effects of genetic risk score (GRS) with lifetime duration of night shift work exposure on the risk of incident COPD (n=62,311).

**Fig. S3.** Joint effects of genetic risk score (GRS) with average lifetime number of night shift work exposure on the risk of incident COPD (n=62,311).

**Table S1. Information on genetic variants associated with COPD**

| **Number** | **rsID** | **Closest gene** | **Locus** | **Risk allele** | **Alt. allele** | **OR** |
| --- | --- | --- | --- | --- | --- | --- |
| 1 | rs13141641 | HHIP | 4q31.21 | T | C | 1.23 |
| 2 | rs17486278 | CHRNA5 | 15q25.1 | C | A | 1.22 |
| 3 | rs7733088 | HTR4 | 5q32 | G | A | 1.18 |
| 4 | rs9399401 | ADGRG6 | 6q24.1 | T | C | 1.14 |
| 5 | rs1441358 | THSD4 | 15q23 | G | T | 1.13 |
| 6 | rs6837671 | FAM13A | 4q22.1 | G | A | 1.16 |
| 7 | rs11727735 | GSTCD | 4q24 | A | G | 1.27 |
| 8 | rs754388 | RIN3 | 14q32.12 | C | G | 1.2 |
| 9 | rs113897301 | ADAM19 | 5q33.3 | AT | A | 1.2 |
| 10 | rs2047409 | TET2 | 4q24 | A | G | 1.1 |
| 11 | rs2955083 | EEFSEC | 3q21.3 | A | T | 1.2 |
| 12 | rs7186831 | CFDP1 | 16q23.1 | A | G | 1.12 |
| 13 | rs10429950 | TGFB2 | 1q41 | T | C | 1.12 |
| 14 | rs2070600 | AGER | 6p21.32 | C | T | 1.28 |
| 15 | rs17707300 | CCDC101 | 16p11.2 | C | T | 1.12 |
| 16 | rs2806356 | ARMC2 | 6q21 | C | T | 1.12 |
| 17 | rs16825267 | PID1 | 2q36.3 | C | G | 1.24 |
| 18 | rs2076295 | DSP | 6p24.3 | T | G | 1.11 |
| 19 | rs647097 | MTCL1 | 18p11.22 | C | T | 1.11 |
| 20 | rs1529672 | RARB | 3p24.2 | C | A | 1.16 |
| 21 | rs721917 | SFTPD | 10q22.3 | G | A | 1.09 |
| 22 | rs12459249 | CYP2A6 | 19q13.2 | C | T | 1.13 |

Abbreviations: COPD, chronic obstructive pulmonary disease; OR, odds ratio.

**Table S2. Basic** **characteristics of participants by lifetime duration of night shift work exposure (n = 74,559)**

| **Characteristics** | **Lifetime duration of night shift work** | | | ***P* value** |
| --- | --- | --- | --- | --- |
|  | **None** | **<10 years** | **≥10 years** |  |
| **No. of subjects (%)** | 56,698 (76.04) | 9,673 (12.97) | 8,188 (10.98) |  |
| Male, No. (%) | 23,693 (41.79) | 5,082 (52.54) | 4,741 (57.90) | <.001 |
| Age, mean (SD), years | 53.05 ± 6.84 | 52.41 ± 6.92 | 52.37 ± 6.70 | <.001 |
| BMI, mean (SD), kg/m^2^ | 26.45 ± 4.47 | 27.16 ± 4.68 | 27.78 ± 4.76 | <.001 |
| White, No. (%) | 55,047 (97.09) | 9,298 (96.12) | 7,842 (95.77) | <.001 |
| Smoking status, No. (%) |  |  |  | <.001 |
| Never | 35,387 (62.41) | 5,315 (54.95) | 4,446 (54.30) |  |
| Previous | 17,527 (30.91) | 3,512 (36.31) | 2,933 (35.82) |  |
| Current | 3,784 (6.67) | 846 (8.75) | 809 (9.88) |  |
| Alcohol drinking, No. (%) |  |  |  | <.001 |
| Never | 1,386 (2.44) | 197 (2.04) | 218 (2.66) |  |
| Previous | 1,248 (2.20) | 256 (2.65) | 228 (2.78) |  |
| Current | 54,064 (95.35) | 9,220 (95.32) | 7,742 (94.55) |  |
| Sleep duration, mean (SD), h/day | 7.09 ± 0.88 | 7.05 ± 0.95 | 7.00 ± 0.97 | <.001 |
| Townsend deprivation index,  median (IQR) | -2.41 (-3.81 to -0.16) | -2.22 (-3.70 to 0.24) | -2.23 (-3.67 to 0.13) | <.001 |
| Asthma, No. (%) | 7,425 (13.10) | 1,323 (13.68) | 1,101 (13.45) | 0.24 |
| IPAQ activity group, No. (%) |  |  |  | <.001 |
| Low | 10,418 (18.37) | 1,662 (17.18) | 1,103 (13.47) |  |
| Moderate | 21,895 (38.62) | 3,450 (35.67) | 2,620 (32.00) |  |
| High | 16,866 (29.75) | 3,381 (34.95) | 3,195 (39.02) |  |
| Not Reported | 7,519 (13.26) | 1,180 (12.20) | 1,270 (15.51) |  |
| Chronotype, No. (%) |  |  |  | <.001 |
| Morning Type | 12,383 (21.84) | 2,127 (21.99) | 1,820 (22.23) |  |
| Intermediate Type | 33,449 (59.00) | 5,678 (58.70) | 4,610 (56.30) |  |
| Evening Type | 4,831 (8.52) | 873 (9.03) | 848 (10.36) |  |
| Not Reported | 6,035 (10.64) | 995 (10.29) | 910 (11.11) |  |

Abbreviations: BMI, body mass index; IPAQ: International Physical Activity Questionnaire.

Values are given as mean ± standard deviation or median (interquartile range) for continuous variables and percentage for categorical variables. Percentages have been rounded and therefore may not total 100.

*P* for values of continuous variables if distributed normally were estimated by one-way ANOVA test, otherwise Kruskal-Wallis test.

*P* for values of categorical variables were estimated by Chi-square test.

**Table S3. Basic characteristics of participants by average lifetime number of night shift work exposure (n = 74,559)**

| **Characteristics** | **Average lifetime night shift frequency** | | | | ***P* value** |
| --- | --- | --- | --- | --- | --- |
|  | **None** | **<3/month** | **3-8/month** | **>8/month** |  |
| **No. of subjects** | 56,698 (76.04) | 2,381 (3.19) | 8,919 (11.96) | 6,561 (8.80) |  |
| Male, No. (%) | 23,693 (41.79) | 1,359 (57.08) | 4,457 (49.97) | 4,007 (61.07) | < .001 |
| Age, mean (SD), years | 53.05 ± 6.84 | 53.00 ± 6.93 | 51.88 ± 6.70 | 52.87 ± 6.89 | < .001 |
| BMI, mean (SD), kg/m^2^ | 26.45 ± 4.47 | 27.15 ± 4.62 | 27.20 ± 4.72 | 27.88 ± 4.73 | < .001 |
| White, No. (%) | 55,047 (97.09) | 2,262 (95.00) | 8,580 (96.20) | 6,298 (95.99) | < .001 |
| Smoking status, No. (%) |  |  |  |  | < .001 |
| Never | 35,387 (62.41) | 1,296 (54.43) | 5,127 (57.48) | 3,338 (50.88) |  |
| Previous | 17,527 (30.91) | 886 (37.21) | 3,053 (34.23) | 2,506 (38.20) |  |
| Current | 3,784 (6.67) | 199 (8.36) | 739 (8.29) | 717 (10.93) |  |
| Alcohol drinking, No. (%) |  |  |  |  | < .001 |
| Never | 1,386 (2.44) | 55 (2.31) | 212 (2.38) | 148 (2.26) |  |
| Previous | 1,248 (2.20) | 81 (3.40) | 211 (2.37) | 192 (2.93) |  |
| Current | 54,064 (95.35) | 2,245 (94.29) | 8,496 (95.26) | 6,221 (94.82) |  |
| Sleep duration,  mean (SD), h/day | 7.09 ± 0.88 | 7.05 ± 0.92 | 7.05 ± 0.94 | 6.97 ± 1.00 | < .001 |
| Townsend deprivation index,  median (IQR) | -2.41 (-3.81 to -0.16) | -2.34 (-3.66 to 0.05) | -2.32 (-3.75 to -0.02) | -2.04 (-3.61 to 0.54) | < .001 |
| Asthma, No. (%) | 7,425 (13.10) | 315 (13.23) | 1,195 (13.40) | 914 (13.93) | 0.27 |
| IPAQ activity group, No. (%) |  |  |  |  | < .001 |
| Low | 10,418 (18.37) | 401 (16.84) | 1,381 (15.48) | 983 (14.98) |  |
| Moderate | 21,895 (38.62) | 824 (34.61) | 3,111 (34.88) | 2,135 (32.54) |  |
| High | 16,866 (29.75) | 820 (34.44) | 3,221 (36.11) | 2,535 (38.64) |  |
| Not Reported | 7,519 (13.26) | 336 (14.11) | 1,206 (13.52) | 908 (13.84) |  |
| Chronotype, No. (%) |  |  |  |  | < .001 |
| Morning Type | 33,449 (59.00) | 1,384 (58.13) | 5,197 (58.27) | 3,707 (56.50) |  |
| Intermediate Type | 12,383 (21.84) | 519 (21.80) | 1,947 (21.83) | 1,481 (22.57) |  |
| Evening Type | 4,831 (8.52) | 217 (9.11) | 821 (9.21) | 683 (10.41) |  |
| Not Reported | 6,035 (10.64) | 261 (10.96) | 954 (10.70) | 690 (10.52) |  |

Abbreviations: BMI, body mass index; IPAQ: International Physical Activity Questionnaire.

Values are given as mean ± standard deviation or median (interquartile range) for continuous variables and percentage for categorical variables. Percentages have been rounded and therefore may not total 100.

*P* for values of continuous variables if distributed normally were estimated by one-way ANOVA test, otherwise Kruskal-Wallis test.

**Table S4. Basic characteristics between all subjects and those with genetic data**

| **Characteristics** | **Current work schedule** | |
| --- | --- | --- |
|  | **Total** | **With genetic data** |
| **No. of subjects (%)** | 277,059 (100.00) | 222,909 (80.46) |
| Male, No. (%) | 133,063 (48.03) | 108,078 (48.49) |
| Age,­­ years | 52.71 ± 7.08 | 52.91 ± 7.06 |
| BMI, mean (SD), kg/m^2^ | 27.24 ± 4.70 | 27.23 ± 4.67 |
| White, No. (%) | 260,152 (93.90) | 222,909 (100.00) |
| Smoking status, No. (%) |  |  |
| Never | 159,908 (57.72) | 128,651 (57.71) |
| Previous | 87,925 (31.74) | 71,525 (32.09) |
| Current | 29,226 (10.55) | 22,733 (10.20) |
| Alcohol drinking, No. (%) |  |  |
| Never | 9,414 (3.40) | 4,873 (2.19) |
| Previous | 7,461 (2.69) | 5,621 (2.52) |
| Current | 260,184 (93.91) | 212,415 (95.29) |
| Sleep duration, mean (SD), h/day | 7.05 ± 0.97 | 7.06 ± 0.95 |
| Townsend deprivation index, median (IQR) | -2.12 (-3.63 to 0.44) | -2.32 (-3.73 to 0.01) |
| Asthma, No. (%) | 36,344 (13.12) | 29,221 (13.11) |
| IPAQ activity group, No. (%) |  |  |
| Low | 45,432 (16.40) | 36,564 (16.40) |
| Moderate | 91,905 (33.17) | 74,455 (33.40) |
| High | 91,350 (32.97) | 74,410 (33.38) |
| Not Reported | 48,372 (17.46) | 37,480 (16.81) |
| Chronotype, No. (%) |  |  |
| Morning Type | 64,189 (23.17) | 51,062 (22.91) |
| Intermediate Type | 159,601 (57.61) | 131,161 (58.84) |
| Evening Type | 22,865 (8.25) | 17,707 (7.94) |
| Not Reported | 30,404 (10.97) | 22,979 (10.31) |

Abbreviations: BMI, body mass index; IPAQ: International Physical Activity Questionnaire.

Values are given as mean ± standard deviation or median (interquartile range) for continuous variables and percentage for categorical variables. Percentages have been rounded and therefore may not total 100.

**Table S5. Adjusted HRs (95% CIs) of the risk of incident COPD by current work schedule and lifetime night shift work experience after excluding participants with COPD occurred within 1 year of follow-up**

| **Variables** | **HR (95% CI)** | ***P* for**  **trend** |
| --- | --- | --- |
| Current work schedule (n=277,057) |  |  |
| Day workers | 1 [Reference] | <.001 |
| Shift, but rarely/some night shifts | 1.28 (1.20, 1.36) |  |
| Usual/permanent night shifts | 1.49 (1.35, 1.66) |  |
| Lifetime duration of night shift work (n=74,559) ^a^ |  | .007 |
| None | 1 [Reference] |  |
| <10 years | 1.17 (1.00, 1.38) |  |
| ≥10 years | 1.23 (1.03, 1.46) |  |
| Average lifetime night shift frequency (n=74,559) ^a^ |  | .001 |
| None | 1 [Reference] |  |
| <3/month | 1.29 (0.97, 1.73) |  |
| 3-8/month | 0.98 (0.81, 1.18) |  |
| >8/month | 1.42 (1.19, 1.68) |  |

Abbreviations: COPD, chronic obstructive pulmonary disease; BMI, body mass index; IPAQ: International Physical Activity Questionnaire.

^a^: The number of subjects is the same as the main analysis because of no participant was excluded from the lifetime night shift work group in this analysis.

Adjusted for age, sex, ethnicity, BMI, Townsend deprivation index, sleep duration, smoking status, alcohol drinking, IPAQ activity group, chronotype, and asthma.

**Table S6. Adjusted HRs (95% CIs) of the risk of incident COPD by current work schedule and lifetime night shift work experience after excluding participants with asthma**

| **Variables** | **HR (95% CI)** | ***P* for**  **trend** |
| --- | --- | --- |
| Current work schedule (n=240,715) |  |  |
| Day workers | 1 [Reference] | <.001 |
| Shift, but rarely/some night shifts | 1.31 (1.20, 1.41) |  |
| Usual/permanent night shifts | 1.49 (1.31, 1.69) |  |
| Lifetime duration of night shift work (n=64,710) |  | 0.02 |
| None | 1 [Reference] |  |
| <10 years | 1.15 (0.94, 1.42) |  |
| ≥10 years | 1.26 (1.02, 1.55) |  |
| Average lifetime night shift frequency (n=64,710) |  | .007 |
| None | 1 [Reference] |  |
| <3/month | 1.22 (0.85, 1.77) |  |
| 3-8/month | 0.99 (0.78, 1.25) |  |
| >8/month | 1.43 (1.16, 1.76) |  |

Abbreviations: COPD, chronic obstructive pulmonary disease; BMI, body mass index; IPAQ: International Physical Activity Questionnaire.

Adjusted for age, sex, ethnicity, BMI, Townsend deprivation index, sleep duration, smoking status, alcohol drinking, IPAQ activity group, and chronotype.

**Table S7. Adjusted HRs (95% CIs) of the risk of incident COPD by current work schedule and lifetime night shift work experience after excluding participants with all missing covariates**

| **Variables** | **HR (95% CI)** | ***P* for**  **trend** |
| --- | --- | --- |
| Current work schedule (n=207,384) |  |  |
| Day workers | 1 [Reference] | <.001 |
| Shift, but rarely/some night shifts | 1.34 (1.24, 1.45) |  |
| Usual/permanent night shifts | 1.49 (1.31, 1.70) |  |
| Lifetime duration of night shift work (n=58,448) |  | .04 |
| None | 1 [Reference] |  |
| <10 years | 1.11 (0.92, 1.34) |  |
| ≥10 years | 1.21 (0.99, 1.48) |  |
| Average lifetime night shift frequency (n=58,448) |  | .02 |
| None | 1 [Reference] |  |
| <3/month | 1.18 (0.84, 1.67) |  |
| 3-8/month | 0.93 (0.75, 1.17) |  |
| >8/month | 1.39 (1.15, 1.70) |  |

Abbreviations: COPD, chronic obstructive pulmonary disease; BMI, body mass index; IPAQ: International Physical Activity Questionnaire.

Adjusted for age, sex, ethnicity, BMI, Townsend deprivation index, sleep duration, smoking status, alcohol drinking, IPAQ activity group, and chronotype.

**Table S8. Associations of current shift work schedule and the risk of incident COPD stratified by sex, age, BMI, and chronotype**

| **Variables** | | **Current work schedule** | | | ***P* for**  **interaction** |
| --- | --- | --- | --- | --- | --- |
|  |  | **Day workers** | **Shift, but rarely/some**  **night shifts** | **Usual/permanent**  **night shifts** |  |
| Sex | |  |  |  | .06 |
|  | Male | 1 [Reference] | 1.31 (1.20, 1.42) | 1.38 (1.21, 1.58) |  |
|  | Female | 1 [Reference] | 1.24 (1.13, 1.37) | 1.70 (1.45, 2.01) |  |
| Age | |  |  |  | .44 |
|  | < 60 years | 1 [Reference] | 1.24 (1.14, 1.35) | 1.36 (1.19, 1.55) |  |
|  | ≥ 60 years | 1 [Reference] | 1.25 (1.12, 1.39) | 1.52 (1.28, 1.81) |  |
| BMI | |  |  |  | .13 |
|  | <25 kg/m^2^ | 1 [Reference] | 1.28 (1.14, 1.45) | 1.65 (1.36, 2.01) |  |
|  | ≥25 kg/m^2^ | 1 [Reference] | 1.30 (1.20, 1.40) | 1.48 (1.31, 1.67) |  |
| Chronotype ^a^ | |  |  |  | .45 |
|  | Moring type | 1 [Reference] | 1.31 (1.15, 1.49) | 1.34 (1.05, 1.72) |  |
|  | Intermediate type | 1 [Reference] | 1.35 (1.24, 1.47) | 1.59 (1.37, 1.84) |  |
|  | Evening type | 1 [Reference] | 1.16 (0.94, 1.43) | 1.52 (1.20, 1.93) |  |

Abbreviations: COPD, chronic obstructive pulmonary disease; BMI, body mass index; IPAQ: International Physical Activity Questionnaire.

Model adjusted for age, sex, BMI, Townsend deprivation index, sleep duration, smoking status, alcohol drinking, IPAQ activity group, chronotype, and asthma.

^a^: Participants in “not reported” group were excluded from this analysis.

**Table S9.** **Associations between chronotype and the risk of incident COPD.**

| **Variables** | **HR (95% CI) of COPD by chronotype** | | |
| --- | --- | --- | --- |
|  | **Intermediate type** | **Morning type** | **Evening type** |
| Model 1 | 1 [Reference] | 1.02 (0.96, 1.08) | 1.45 (1.33, 1.57) |
| Model 2 | 1 [Reference] | 1.04 (0.98, 1.10) | 1.12 (1.03, 1.22) |
| Model 3 | 1 [Reference] | 1.03 (0.97, 1.09) | 1.09 (1.01, 1.19) |

Abbreviations: COPD, chronic obstructive pulmonary disease; BMI, body mass index; IPAQ: International Physical Activity Questionnaire.

Model 1: age and sex.

Model 2: Model 1 + ethnicity, BMI, Townsend deprivation index, sleep duration, smoking status, alcohol drinking, IPAQ activity group and shift work.

Model 3: Model 2 + asthma.

**Table S10. Adjusted HRs (95% CIs) of incident COPD by individual genetic risk score (GRS) of participants of current work schedule (n=222,909)**

| **Variables** | **HR (95% CI)**  **per SD increase** | **GRS in quartiles** | | | | ***P* for**  **trend** |
| --- | --- | --- | --- | --- | --- | --- |
|  |  | **Q1** | **Q2** | **Q3** | **Q4** |  |
| Model 1 | 1.09 (1.06, 1.11) | 1 [Reference] | 1.07 (0.99, 1.16) | 1.16 (1.07, 1.25) | 1.24 (1.15, 1.33) | <.001 |
| Model 2 | 1.09 (1.06, 1.12) | 1 [Reference] | 1.08 (0.99, 1.16) | 1.16 (1.07, 1.25) | 1.24 (1.15, 1.34) | <.001 |
| Model 3 | 1.09 (1.06, 1.12) | 1 [Reference] | 1.08 (1.00, 1.16) | 1.16 (1.08, 1.25) | 1.24 (1.15, 1.34) | <.001 |

Abbreviations: HR, hazard ratio; CI, confidence interval; COPD, chronic obstructive pulmonary disease; GRS, genetic risk score.

Model 1: unadjusted.

Model 2: adjusted for age and sex.

Model 3: adjusted for age, sex, and the first 10 principal components of ancestry.

**Figure S1. Association of genetic risk score (GRS) and the risk of incident COPD (n=222,909)**


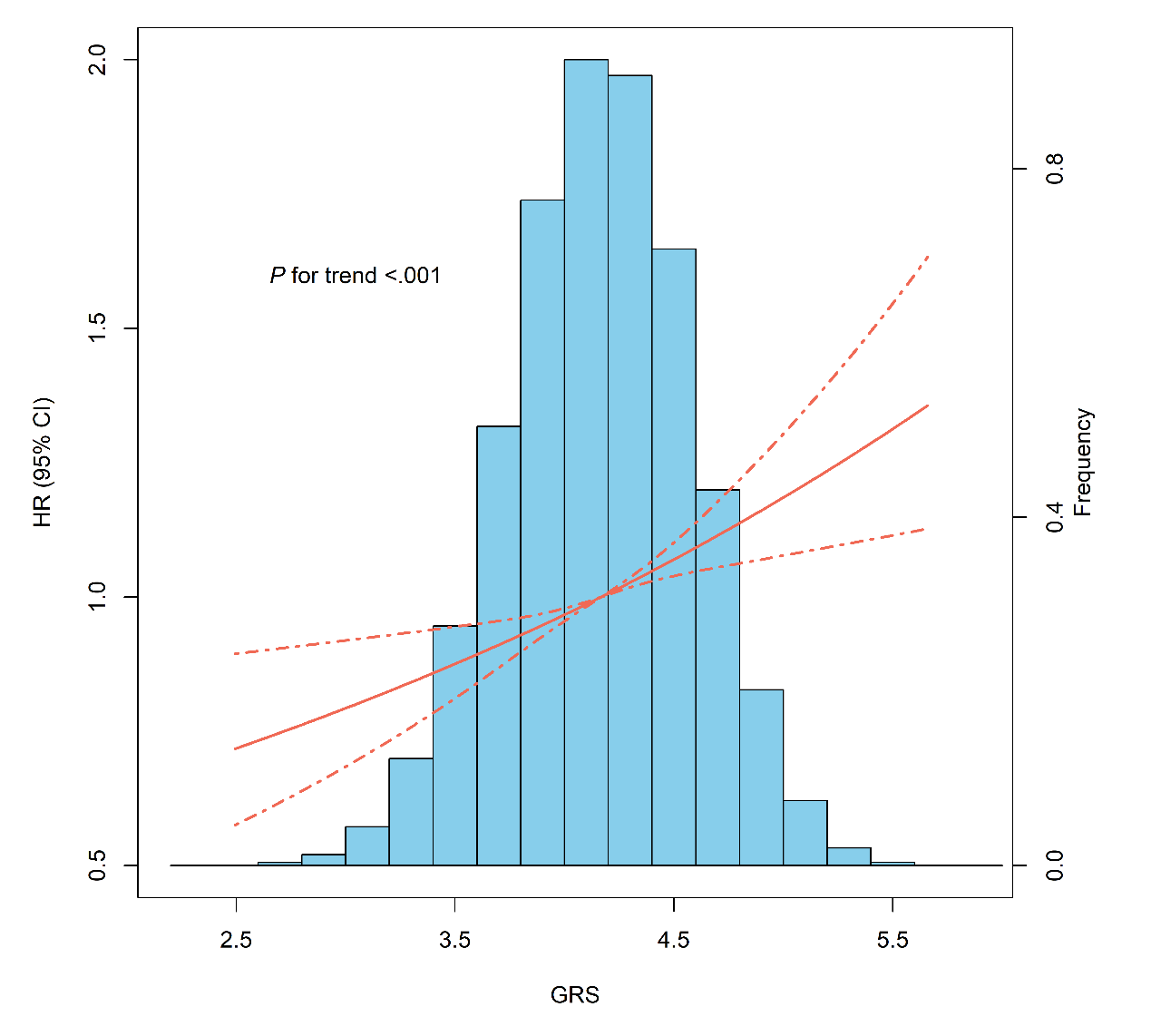


Abbreviations: GRS, genetic risk score; COPD, chronic obstructive pulmonary disease; HR, hazard ratio; CI, confidence interval.

Adjusted for age, sex, and the first 10 principal components of ancestry.

**Figure S2. Joint effects of genetic risk score (GRS) with lifetime duration of night shift work exposure on the risk of incident COPD (n=62,311)**


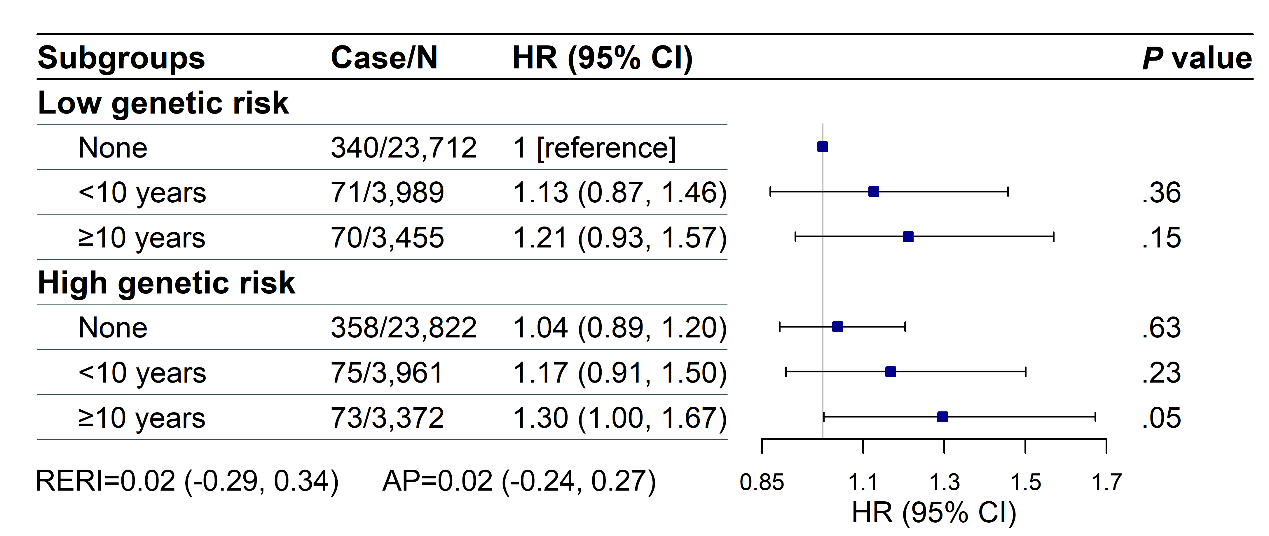


Abbreviations: COPD, chronic obstructive pulmonary disease; HR, hazard ratio; CI, confidence interval; BMI, body mass index; IPAQ: International Physical Activity Questionnaire.

Adjusted for age, sex, BMI, Townsend deprivation index, sleep duration, smoking status, alcohol drinking, IPAQ activity group, chronotype, and asthma.

**Figure S3. Joint effects of genetic risk score (GRS) with average lifetime number of night shift work exposure on the risk of incident COPD (n=62,311)**


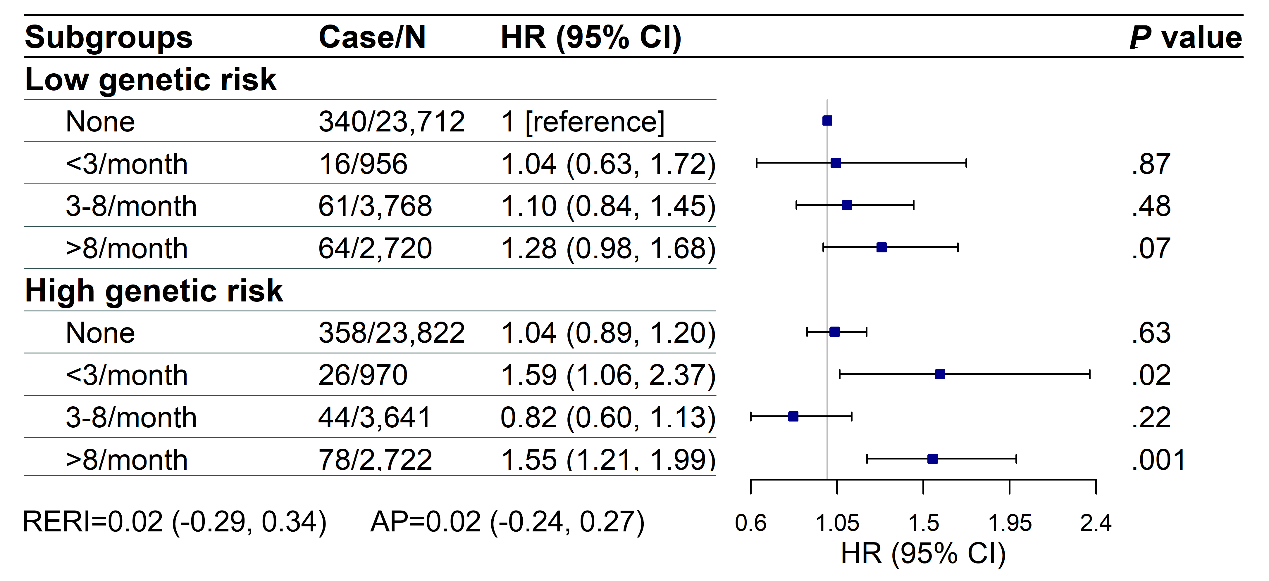


Abbreviations: COPD, chronic obstructive pulmonary disease; HR, hazard ratio; CI, confidence interval; BMI, body mass index; IPAQ: International Physical Activity Questionnaire.

Adjusted for age, sex, BMI, Townsend deprivation index, sleep duration, smoking status, alcohol drinking, IPAQ activity group, chronotype and asthma.
